# Molecular Longitudinal Tracking of *Mycobacterium abscessus* spp. during Chronic Infection of the Human Lung

**DOI:** 10.1371/journal.pone.0063237

**Published:** 2013-05-16

**Authors:** Kaj M. Kreutzfeldt, Paul R. McAdam, Pauline Claxton, Anne Holmes, A. Louise Seagar, Ian F. Laurenson, J. Ross Fitzgerald

**Affiliations:** 1 The Roslin Institute and Edinburgh Infectious Diseases, University of Edinburgh, Easter Bush Campus, Edinburgh, United Kingdom; 2 Scottish Mycobacteria Reference Laboratory (SMRL), Clinical Microbiology, Royal Infirmary of Edinburgh, Little France, Edinburgh, United Kingdom; Hopital Raymond Poincare - Universite Versailles St. Quentin, France

## Abstract

The *Mycobacterium abscessus* complex is an emerging cause of chronic pulmonary infection in patients with underlying lung disease. The *M. abscessus* complex is regarded as an environmental pathogen but its molecular adaptation to the human lung during long-term infection is poorly understood. Here we carried out a longitudinal molecular epidemiological analysis of 178 *M. abscessus* spp. isolates obtained from 10 cystic fibrosis (CF) and 2 non CF patients over a 13 year period. Multi-locus sequence and molecular typing analysis revealed that 11 of 12 patients were persistently colonized with the same genotype during the course of the infection while replacement of a *M. abscessus sensu stricto* strain with a *Mycobacterium massiliense* strain was observed for a single patient. Of note, several patients including a pair of siblings were colonized with closely-related strains consistent with intra-familial transmission or a common infection reservoir. In general, a switch from smooth to rough colony morphology was observed during the course of long-term infection, which in some cases correlated with an increasing severity of clinical symptoms. To examine evolution during long-term infection of the CF lung we compared the genome sequences of 6 sequential isolates of *Mycobacterium bolletii* obtained from a single patient over an 11 year period, revealing a heterogeneous clonal infecting population with mutations in regulators controlling the expression of virulence factors and complex lipids. Taken together, these data provide new insights into the epidemiology of *M. abscessus* spp. during long-term infection of the CF lung, and the molecular transition from saprophytic organism to human pathogen.

## Introduction

The *Mycobacterium abscessus* complex is a group of rapidly growing mycobacteria (RGM) that is associated with an array of human infections typically affecting the lungs, skin or soft tissues [1]–[4]. Chronic lung infection is most frequently observed in patients with underlying lung disease, especially those suffering from cystic fibrosis (CF) [5], [6], and *M. abscessus* spp. infections in CF patients are linked to disease progression [2]. *M. abscessus* complex is subdivided into the 3 species *M. abscessus sensu stricto*, *Mycobacterium massiliense* and *Mycobacterium bolletii* which exhibit clinically relevant differences in antibiotic sensitivity profile [7]–[9].

Chronic infections caused by environmental pathogens are characterized by the ability of the infecting organisms to adapt to a very different niche habitat, leading to changes in phenotype influencing colony morphology, inflammatory response, and antibiotic resistance [10]–[12]. Like other RGM the *M. abscessus* spp. are saprophytic organisms associated with water reservoirs linked to human activity [13], [14]. *M. abscessus sensu stricto* is innately resistant to many classes of antibiotic [15] and readily acquires resistance to clarithromycin during infection [16]. In addition, *M. abscessus sensu stricto* exhibits differences in colony morphology including smooth colony variants typical of environmental and early infection isolates [6] which are replaced over a period of years with rough variants capable of invasion of macrophage and respiratory epithelial cells, and associated with severe inflammation [17], [18]. Previous studies have demonstrated the long-term persistence of single *M. abscessus* spp. strains during chronic infection of the CF lung with occasional sharing of strains between sibling pairs, implying that transmission between patients may occur [19]. A recent study provided evidence for an outbreak involving *M. massiliense* infection of 5 patients attending a CF clinic implying that some strains of *M. abscessus* complex may have epidemic potential [1].

The phenomenon of long-term persistence of *M. abscessus* complex during chronic infection of the human lung is well established. However, the relative prevalence of the 3 *M. abscessus* species among long-term infected patients, and the capacity of *M. abscessus* complex to transmit between patients have not been well examined. Here, we carry out longitudinal molecular tracking of *M. abscessus* complex isolates from chronically infected patients with multi-locus sequence and PCR-based bacterial typing. We also combine whole genome sequencing of sequential isolates with phenotypic analysis of colony morphology and antibiotic sensitivity to examine the adaptive diversification of *M. abscessus* spp. during chronic infection of the human lung revealing a genetically and phenotypically heterogeneous infecting population. In addition, the data provide molecular correlates of adaptation of *M. abscessus* spp. to the human lung that will inform future studies investigating *M. abscessus* pathogenesis.

## Materials and Methods

### *M. abscessus* Complex Isolates and Culture Conditions

All clinical isolates were referred to the Scottish Mycobacteria Reference Laboratory (SMRL) for identification, susceptibility testing, DNA extraction and long-term storage. Ethical approval for use of samples/data was provided by the National Health Service, South East of Scotland HSS BioResource facility operating within the Tissue Act Scotland 2006. The Scientific Officer indicated that the research does not need NHS ethical review under the terms of the Governance Arrangements for Research Ethics Committees (A Harmonised Edition). In particular, research limited to secondary use of information previously collected in the course of normal care (without an intention to use it for research at the time of collection) is generally excluded from REC review, provided that the patients or service users are not identifiable to the research team carrying out the research. The review board waived the need for written informed consent from the participants.

A total of 178 *M. abscessus* spp. pulmonary isolates were obtained from 12 patients in Scotland between 1998 and 2010, including 10 unrelated patients and 1 sibling pair. Annual incidence of *M. abscessus* spp. infection is estimated at ∼887/100,000 in Scottish cystic fibrosis patients or about 8 patients annually (Scottish Mycobacterium Reference laboratory, data not shown). Of the 12 patients, 10 suffered from cystic fibrosis and 2 had significant co-morbidities including cirrhotic alcoholic liver disease, and acute myeloid leukemia, both with chronic pulmonary disease (Table 1). The mean age at first isolation of *M. abscessus* amongst the CF patients was 15 y (range 12–23 y). One CF patient successfully cleared carriage and went on to lung transplantation. Isolates were identified as *M. abscessus* spp. by GenoType Mycobacterium CM test (HAIN Lifescience, Germany). Species type strains included *M. abscessus* NCTC 13031, *M. bolletii* DSM 45149 and *M. massiliense* DSM 45103. Isolates were cultured on Loewenstein Jensen egg slopes or Columbia Horse Blood Agar (Oxoid, Hampshire, UK) statically at 37°C.

**Table 1:** Summary of patient, isolate and clinical information.

			Patient 1	Patient 2	Patient 3	Patient 4	Patient 5	Patient 6
Year of birth			1986	1985	1986	1934	1960	1980
Sex			F	F	F	M	M	F
CF status			CF	CF	CF	Non-CF	Non-CF	CF
Number of isolates			25	23	19	18	14	15
Age in years at first/most recent isolation			13/25	15/21	12/24	71/76	43/46	17/19
Species			*M. bolletii*	*M. massiliense*	*M. abscessus sensu stricto*	*M. abscessus sensu stricto*	*M. abscessus sensu stricto*	*M. massiliense*
Sequence type			119	23	26	120	121	23
ERIC- PCR type			12	3	8	9	10	4
Colony morphology								
	Smooth to rough switch		x	x			x	
	Rough only					x		x
	Mixed smooth and rough		x		x			
Clinical comments			See text	Died aged 21 y	Alive	Acute myeloid leukaemia, COPD, died aged 77 y	Alcoholic liver disease withliver cirrhosis, bronchiectasis,died aged 48 y	Died aged 21 y
			Patient 7	Patient 8	Patient 9	Patient 10	Patient 11	Patient 12
Year of birth			1975	1989	1992	1983	1988	1987
Sex			M	M	F	F	F	F
CF status			CF	CF	CF	CF	CF	CF
Number of isolates			15	12	12	10	9	6
Age in years at first/most recent isolation			23/29	10/16	12/14	19/25	16/21	13/15
Species			*M. massiliense*	*M. massiliense*	*M. abscessus sensu* *stricto/M. massiliense*	*M. abscessus sensu stricto*	*M. bolletii*	*M. massiliense*
Sequence type			69	23	122/23	123	124	23
ERIC-PCR type			5	4	7/2	6	11	1
Colony morphology								
		Smooth to rough switch		x	x			x
		Rough only	x		x	x	x	
		Mixed smooth and rough						
Clinical comments			Died aged 32 y	Died aged 21 y	Died aged 14 y	Alive	Died aged 21 y	Cleared infection, lung transplant aged 17 y

### Genomic DNA Extraction

Genomic DNA (gDNA) was extracted from bacteria following the method described by van Soolingen et al. [20] and previously used for *M. abscessus* spp. [21]. DNA yield was quantified using a Nanodrop ND-1000 Spectrophotometer (Thermo Scientific, USA).

### ERIC-PCR Typing

For ERIC-PCR, 50 µl reactions included 2 µM forward and reverse oligonucleotide primers described by Sechi et al. [22], 0.25 U of Go-Taq Flexi DNA Polymerase, 5x Green Go Taq Flexi Buffer, 1.5 mM MgCl_2_, 200 µM dNTPs (Promega, Hampshire, UK) and 10 ng approx. gDNA. Thermocycling conditions were 3 min at 95°C followed by 30 cycles of 95°C for 1 min, 55°C for 1 min and 72°C for 5 min with a final extension of 10 min at 72°C. Agarose gel electrophoresis of ERIC-PCR products was followed by comparative analysis of resolved profiles with the BioNumerics program v5 with clustering by UPGMA with Dice similarity. Independent genomic DNA extractions resulted in indistinguishable ERIC-PCR profiles, demonstrating the method to be highly reproducible (data not shown).

### Multi-locus Sequence Typing (MLST)

MLST was carried out as described previously [9]. Briefly, 50 µl PCR reactions specific for the 7 housekeeping loci included 0.2 µM forward and reverse oligonucleotide primers, 0.25 U of Go-Taq Flexi DNA Polymerase, 5x Green Go Taq Flexi Buffer, 1.5 mM MgCl_2_, 200 µM dNTPs (Promega, Hampshire, UK) and 10 ng approx. gDNA. Thermocycling conditions were 3 min at 95°C followed by 30 cycles of 95°C for 1 min, 55°–60°C for 1 min and 72°C for 1 min with a final extension of 10 min at 72°C. Sequencing was carried out by The GenePool Sequencing Service (King’s Buildings, University of Edinburgh, UK), and sequences assembled using Geneious 5.4.2. [23]. Alleles and sequence types were determined using the Institute Pasteur *M. abscessus* MLST database (http://www.pasteur.fr/recherche/genopole/PF8/mlst/references_abscessus.html). Single and multi-gene phylogenies were re-constructed using a neighbor-joining method with the HKY model of nucleotide substitution, with support for nodes assessed by 1000 bootstrap replicates.

### Whole Genome Sequencing and Analysis

Whole genome sequencing was carried out with the Illumina GA2 sequencing platform. The FASTX toolkit [24] was used to assess read quality, remove adaptor sequences, remove 3 bp from the 5′ end of the read and filter reads so that at least 80% of bases within a read were of PHRED score Q30 or above. Paired-end reads were mapped to a draft genome sequence of *M. bolletii* [25] using the BWA short read aligner [26]. Polymorphic sites were identified at loci covered by at least 3 reads where the alternative base was present in at least two thirds of the reads with PHRED equivalent score greater than 30, and inspected manually. As an additional confirmation, reads were mapped to the genome of *M. abscessus sensu stricto* strain ATCC 19977 (accession number CU458896), and only those variants present in both reference genomes are reported. A neighbor-joining phylogeny was reconstructed based on the polymorphic sites identified using the HKY substitution model. 1000 bootstrap replicates were used to determine support for key nodes.

### Antibiotic Susceptibility Testing

MICs of amikacin, amoxicillin/clavulanic acid, cefepime, cefoxitin, ceftriaxone, ciprofloxacin, clarithromycin, doxycycline, imipenem, linezolid, minocycline, moxifloxacin, tigecycline, tobramycin, trimethoprim/sulphametoxazole were determined by the Clinical and Laboratory Standards Institute recommended micro-dilution method in Mueller-Hinton broth using Sensititre RGMYCO plates (TREK Diagostics, USA). Plates were incubated at 37°C and successive readings were taken after 5, 6, 8, 9, 12 and 14 d of incubation.

## Results and Discussion

### Molecular Population Genetic Analysis of *M. abscessus* spp. from Chronically Infected Patients

**Figure 1:**
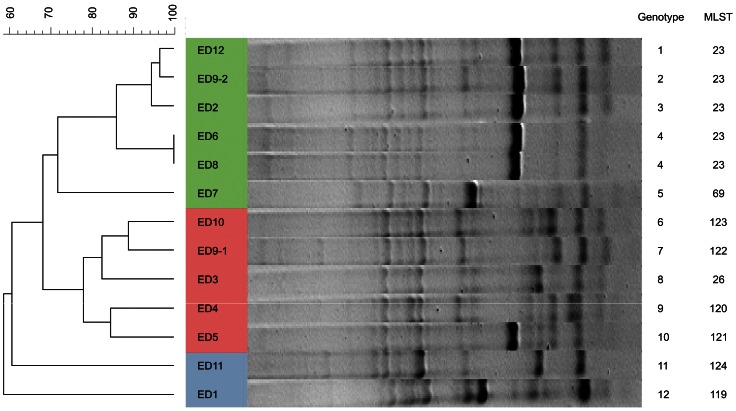
Genotypic analysis of *M.* abscessus spp. isolates from persistently infected patients. Dendrogram constructed using BioNumerics showing the genetic relatedness among ERIC-PCR profiles of 1 representative isolates from each of the 12 patients.

In order to examine the long-term persistence of *M. abscessus* spp. during infection of the human lung, we analyzed a total of 178 sequential *M. abscessus* complex isolates from 12 patients and carried out multi-locus sequence type (MLST) analysis of each unique ERIC-PCR genotype identified in each patient. ERIC-PCR has been shown to be highly discriminatory for grouping outbreak strains of *M. abscessus* spp. and does not suffer from problems of degradation that occur with some electrophoresis-based methods [21]. In total, 12 genotypes representing 9 distinct sequence types (ST), including 6 novel STs and 3 *M. abscessus* complex species, were identified among the 12 patients (Fig. 1; Table 1). For 11 of 12 patients, a single genotype was identified during long-term infection (2 to 12 years) consistent with persistent infection with the same strain. In contrast, for patient 9, the first 5 chronological isolates exhibited a genotype that was distinct from that of the subsequent seven isolates suggesting replacement of the original infecting strain during the course of infection (Fig. 1). MLST analysis of the isolates from patient 9 revealed that an *M. abscessus sensu stricto* strain was replaced by an *M. massiliense* strain (Table 1). Our findings are in agreement with other studies that compared sequential *M. abscessus* isolates from CF patients highlighting the long-term persistence of *M. abscessus*, and its treatment-refractory nature [6], [27], [28]. However, the data indicate for the first time that replacement of *M. abscessus* by unrelated strains may occur during chronic infection.

### *M. massiliense* ST23 is Prevalent among CF Patients

**Figure 2:**
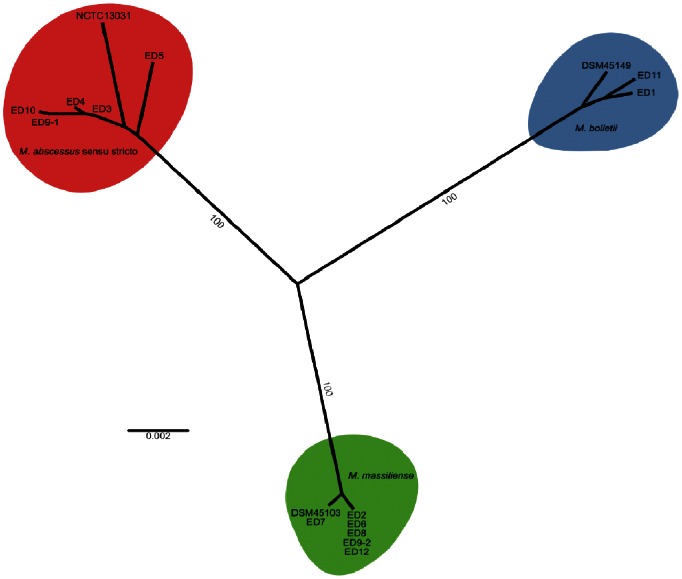
Phylogenetic reconstruction (MLST) resolves the species designation of each *M.* abscessus spp. isolate. Neighbour-joining tree of 13 clinical isolates and type strains *M. abscessus* NCTC 13031, *M. bolletii* DSM 45149 and *M. massiliense* DSM 45103 based on four concatenated MLST gene sequences (*argH, glpK, gnd* and *murC*). Colour coding indicates *M. abscessus sensu stricto* (red), *M. bolletii* (blue) and *M. massiliense* (green)-associated clades. Bootstrap support for significant branches is indicated, and branch lengths are representative of substitutions per site.

Single gene trees based on *argH, glpK, gnd* or *murC* sequences exhibited 3 distinct clades that co-segregate with the type strains *M. abscessus* NCTC 13031, *M. massiliense* DSM 45103 and *M. bolletii* DSM 45149 (Fig. S1 in File S1). In contrast, the trees constructed with *cya, pta and purH* sequences have topologies that imply horizontal transfer of these alleles among the *M. abscessus* complex. Of note, substantial horizontal gene transfer between members of the *M. abscessus* complex has previously been demonstrated [9]. Phylogenetic reconstruction based on the concatenated sequences of the 4 MLST loci that did not display evidence of horizontal transfer resulted in a tree with 3 well-supported clades, each including one of the *M. abscessus* species type strains (Fig. 2), facilitating species designation for the clinical isolates. Of the 12 patients, 4 were infected with *M. abscessus sensu stricto*, 5 with *M. massiliense*, 2 with *M. bolletii* and 1 (patient 9) was infected with *M. abscessus sensu stricto* initially followed by replacement with *M. massiliense* (Fig. 2; Table 1). Although a number of studies have reported a higher prevalence of *M. abscessus sensu stricto* among clinical isolates [8], [29], Zelazny et al. reported that *M. massiliense* was most frequently isolated from the airways of young patients with an underlying lung disease [30]. Notably, 5 of 6 *M. massiliense* isolates belonged to ST23 which is highly prevalent in the *M. abscessus* MLST database and has been reported in France, Germany, Switzerland and an outbreak of skin and soft tissue infections in Brazil. The finding that a single sequence type is associated with disease in distant countries is noteworthy and could indicate limited clonal variation among *M. massiliense* strains. Alternatively, ST23 may represent a *M. massiliense* subtype with enhanced pathogenic potential, or the capacity to resist aggressive antibiotic treatment.

In the current study, isolates (ED2 and ED12) obtained from a pair of siblings had closely-related genotypes of the same ST23, consistent with a common infective source or cross infection (Fig. 1). Of note, a recent study reports the likely transmission of *M. abscessus* spp. between patients [1]. Furthermore, *M. abscessus* spp. have been reported to occur in hospital water distribution systems and clusters of infections have previously been linked to contaminated bronchoscopes [31], [32]. Huang et al. typed *M. abscessus* spp. isolates from clinical and environmental sources linking some infections to *M. abscessus* spp. present in water distribution systems but the possibility of patient-to-patient transmission could not be excluded [33].

### Transition from Smooth to Rough Colony Morphology during Chronic *M. abscessus* spp. Infection

Previous studies have reported a correlation between a switch in smooth to rough colony morphology and an increase in virulence [34]. In order to determine if *M. abscessus* spp. undergo changes in colony morphology during long-term infection, the colony phenotype of all isolates was examined. In 5 patients, the majority of earlier isolates in these patients had a smooth colony morphology with a small number of isolates demonstrating a rough colony phenotype. However, over several years of persistent infection, there was an increase in the proportion of isolates with a rough colony morphology leading to uniformly rough colony morphology for the later stage isolates (Table 1, Table S2 from File S2). From patient 1, persistently infected with *M. bolletii*, 21 of 25 isolates obtained between 1998 and 2005 demonstrated raised, moist, smooth colonies. The remaining four isolates, all collected between 2006 and 2009, contained a mixture of smooth and rough colonies. The timing of the emergence of rough colony phenotype isolates from patient 1 correlates with an increase in severity of clinical disease that was observed in 2005. The transition in colony phenotype has been linked to an increase in severity of infection as the rough phenotype induces an elevated inflammatory response in the host [34]. It is feasible that in the patients with rough colony isolates only, the *M. abscessus* infection was asymptomatic until a smooth to rough colony phenotype switch resulted in elevated inflammation and clinical symptoms.

### Susceptibility of *M. bolletii* to Antibiotics during Long-term Infection of a Single Patient

**Figure 3:**
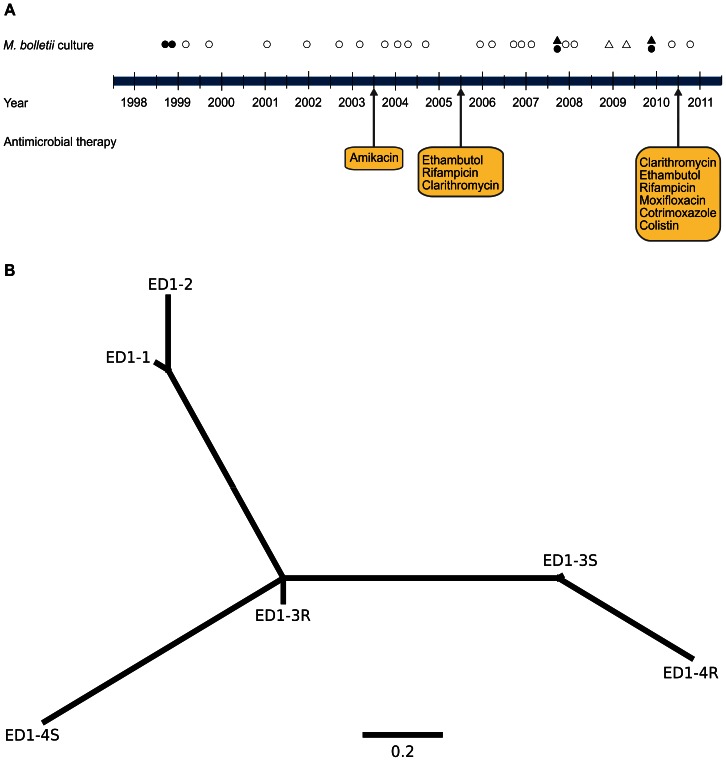
Evolutionary genomic analysis of *M.* bolletii during a 12 year chronic infection of a single CF patient. (A) Schematic representation of the timescale of infection including isolation dates of smooth colony phenotype (circles), rough colony phenotype (triangles), and sequenced isolates (filled symbols), respectively. Approximate time periods of antibiotic treatment are indicated by arrows. (B) Neighbor-joining phylogenetic tree of *M. bolletii* isolates from a single patient based on core genome SNPs.

In order to examine the antibiotic sensitivity of *M. abscessus* spp. isolates during long term infection of the CF lung in a patient undergoing defined antibiotic treatment regimens, we examined 6 *M. bolletii* isolates obtained sequentially from a single patient (Patient 1) over a 12 year period. She had received multiple courses of antimicrobials over 12 years following deterioration in her pulmonary function including a combination of ethambutol, rifampicin and clarithromycin, and had previously been treated with amikacin (Fig. 3). The patient was turned down for lung transplantation in view of *M. abscessus* spp. culture-positivity. Subsequently, the patient was treated with nebulized interferon gamma but was again turned down for transplantation due to extensive pleural disease. In June 2011, she had advanced cystic fibrosis requiring ambulatory oxygen and was taking ethambutol, rifampicin, clarithromycin, moxifloxacin, cotrimoxazole and nebulised colistin. Amoxicillin/clavulanic acid, cefepime, ciprofloxacin, doxycycline, minocycline, moxifloxacin, tobramycin and trimethoprim/sulphamethoxazole demonstrated no activity against any of the isolates tested (data not shown). All isolates were found to be resistant to clarithromycin with MICs of >16 µg/ml after 14 days of incubation and 5 out of 6 isolates were resistant to clarithromycin with an MIC of 8 µg/ml from day 5 (Table S1 in File S1). *M. bolletii* encodes the erythromycin ribosome methyltransferase (*erm)* determinant which facilitates inducible resistance to clarithromycin [35]. The observed resistance from as early as day 5 of reading susceptibility results is consistent with Patient 1 having received clarithromycin since 2005, with intermittent macrolide treatment prior to that. The isolates tested demonstrated intermediate resistance to amikacin and were fully resistant to cefoxitin and ciprofloxacin. Of note, this patient received treatment with amikacin prior to 2005, which might explain the reduced sensitivity to this antibiotic. All 3 *M. abscessus* spp. type strains demonstrated sensitivity to amikacin (data not shown). A previous study reported that all *M. bolletii* isolates exhibited high drug resistance to ciprofloxacin and cefoxitin with intermediate resistance or resistance to amikacin [36]. However *M. bolletii* isolates susceptible to amikacin and with intermediate resistance to cefoxitin have also been reported in the literature [35]. Taken together, these data demonstrate the high levels of innate *in vitro* antibiotic resistance among *M. bolletii* clinical isolates.

### Genome-wide Examination of *M. bolletii* Adaptation to the Human CF Lung

To investigate the molecular adaptation of *M. abscessus* spp. to the human lung, we determined the whole genome sequence of 6 sequential isolates of *M. bolletii* obtained over a 12 year period from a single persistently infected patient (Table 2). Isolates were selected to represent the temporal and phenotypic diversity of the isolates obtained from patient 1 (Table 2). The core genome of the 6 strains, identified as all shared nucleotide sites, was comprised of 4,178,261 sites, and we identified 14,594 variable sites with respect to the reference genome of *M. bolletii* type strain CIP108541 [25]. A total number of 34 SNPs (median coverage 21 reads) and 5 nucleotide insertions or deletions were identified as variable within the core genome among the 6 sequenced genomes (Table 3). A neighbor-joining tree based on the SNP differences identified among the 6 isolates consisted of 3 clades with the 2 isolates obtained in 1998 represented on the same branch (Fig. 3). However, isolates from different years were more closely related to each other than to isolates obtained in the same year indicating the existence of a heterogeneous infecting population containing multiple persisting sub-lineages (Fig. 3). The diversification of bacterial pathogens in the CF lung leading to heterogeneous infecting populations has been previously described for *Burkholderia dolosa*, *Pseudomonas aeruginosa* and *Staphylococcus aureus* [12], [37], [38].

**Table 2:** Isolates from patient 1 selected for whole genome sequencing.

	Specimendate	Colony morphology	MIC Clarithromycin (mg/l)
ED1-1	1998	Smooth	>16
ED1-2	1998	Smooth	>16
ED1-3S*	2006	Smooth	>16
ED1-3R*	2006	Rough	>16
ED1-4S*	2009	Smooth	>16
ED1-4R*	2009	Rough	>16

* Isolates 3S/R and 4S/R were obtained from the same specimen.

**Table 3:** Genome variation among sequential *M. bolletii* isolates from a single patient.

	*Type of mutation in isolates:										
Gene Product	ED1-1	ED1-2		ED1-3R		ED1-3S		ED1-4R		ED1-4S	
Arabinosyltransferase C				NS		NS		NS		NS	
Prephenate dehydrogenase										NS	
Amine oxidase				NS							
Glutamate-cysteine ligase GshA				NS		NS		NS		NS	
Sensor histidine kinase PhoR						NS		NS		NS	
Putative YrbE family protein										NS	
Fatty-acid-CoA ligase FadD										NS	
Putative alkanesulfonate monooxygenase						STOP		STOP			
Oxidoreductase								S			
Indole-3-glycerol-phosphate synthase										NS	
Acetolactate synthase										S	
50S ribosomal protein L14				NS							
Ribonuclease III						NS		NS			
Cytochrome P450	NS										
Glycosyltransferase GtfA				NS							
	* **Type of mutation in isolates:**										
**Gene Product**	**ED1-1**	**ED1-2**		**ED1-3R**		**ED1-3S**		**ED1-4R**		**ED1-4S**	
Hydrogen peroxide-inducible activator						NS		NS			
Putative monooxygenase								NS			
Luciferase-like monooxygenase										S	
Oxalate decarboxylase		NS									
PPE family protein						NS		NS			
23S ribosomal RNA						NS					
Transcriptional regulatory protein								NS			
Transcriptional regulatory protein				S		S		S		S	
Hypothetical protein				NS							
Hypothetical protein		STOP									
Hypothetical protein								S			
Intergenic				NCS		NCS		NCS		NCS	
Intergenic				NCS		NCS		NCS		NCS	
Intergenic						NCS		NCS			
Intergenic										NCS	
Intergenic								NCS			
Intergenic						NCS		NCS			
	* **Type of mutation in isolates:**										
**Gene Product**	**ED1-1**		**ED1-2**		**ED1-3R**		**ED1-3S**		**ED1-4R**		**ED1-4S**
Hypothetical protein							FSD		FSD		
Hypothetical protein					FSD						
Putative aminotransferase									FSI		
Hypothetical protein							FSI				
Intergenic					NCI		NCI		NCI		NCI

* NS, non-synonymous SNP; S, synonymous SNP; NCS, non-coding SNP; FSI, frameshift insertion; FSD, frameshift deletion; NCI, non-coding insertion.

Of the 34 SNPs identified among the 6 isolates, 24 gave rise to non-synonymous mutations (Table 3). Of note, the *phoR* gene was affected by 2 distinct non-synonymous mutations in 3 out of the 6 isolates (Table 3; Table S3 in File S2). In *Mycobacterium tuberculosis* PhoR is the histidine kinase component of the two-component system PhoPR involved in the biosynthesis of complex lipids required for growth of *M. tuberculosis* inside macrophages and mice [39]. The occurrence of multiple independent mutations of *phoR* implies that *phoR* attenuation may confer a selective advantage in the CF lung. In the ED1-4R isolate, a large deletion of the *mmpl* gene which encodes Mmpl, a putative membrane protein, was identified. Deletion of *mmpL4b* has been demonstrated to result in a switch from smooth to rough phenotype [40], [41] suggesting that this deletion may be responsible for the rough phenotype of the isolate. Furthermore, several non-synonymous mutations were found in genes that are involved in bacterial metabolism, and regulation of gene expression, leading us to speculate that they may contribute to the radical transition from a saprophytic to human pathogenic lifestyle. Finally, the acquisition of amikacin resistance by *M. bolletii* relative to the amikacin-sensitive reference strain led us to examine the genome sequence for the underlying mutations responsible. Previous studies have demonstrated that polymorphisms in the 16S rRNA of *M. tuberculosis* result in resistance to amikacin [42]. However, there were no polymorphisms identified in the 16S rRNA of the amikacin-resistant *M. bolletii* isolates, suggesting that an alternative mechanism of amikacin resistance may exist in *M. bolletii*.

### Concluding Comments

*M. abscessus* spp. have emerged as an important cause of pulmonary infection among CF patients with the capacity for long-term persistence leading to clinical deterioration over time. Our genetic typing analysis demonstrates the capacity of *M. abscessus* spp. isolates to persist for at least 12 years during long-term infection but also indicates that replacement of *M. abscessus* spp. can occur occasionally during long-term infection. The study provides an indication of the relative prevalence of the 3 *M. abscessus* species among a selection of infected patients in Scotland and reveals that a single globally distributed ST of *M. massiliense* (ST23) is also prevalent among Scottish patients. An investigation into the origin and pathogenic capacity of ST23 is warranted to understand the basis for the existence of a widespread *M. massiliense* clone among infected patients. Finally, the first genome-wide analysis of the evolution of *M. abscessus* spp. during long term infection revealed the heterogeneous nature of the infective *M. bolletii* population, and led to the identification of mutations which may contribute to the adaptation from saprophytic organism to human pathogen. Characterization of the genetic determinants involved in the transition of *M. abscessus* spp. from initial colonization to severe lung pathology could lead to the identification of novel drug targets for the control of *M. abscessus* spp. infections.

## Supporting Information

File S1 **This supporting file includes Figure S1 and Table S1.** Figure S1: Phylogenetic analysis of *M. abscessus* spp. isolates based on single MLST loci. Table S1: Clarithromycin, Cefoxitin and Amikacin MICs determined for isolates from Patient 1.
(PDF)

File S2 **This supporting file includes Table S2 and Table S3.** Table S2: Genetic and phenotypic characteristics of each of the 178 study isolates. Table S3: Genome variation among sequential *M. bolletii* isolates from a single patient.
(XLS)
