# Supplementary material for: Molecular Longitudinal Tracking of Mycobacterium abscessus spp. during Chronic Infection of the Human Lung
Source: PLoS One. 2013 May 16;8(5):e63237. doi: 10.1371/journal.pone.0063237 (PMC3655965; doi:10.1371/journal.pone.0063237)
Supplement: File S1 — This supporting file includes Figure S1 and Table S1. Figure S1: Phylogenetic analysis of M. abscessus spp. isolates based on single MLST loci. Table S1: Clarithromycin, Cefoxitin and Amikacin MICs determined for isolates from Patient 1. (PDF) [file pone.0063237.s001.pdf]

Table S1. Clarithromycin, Cefoxitin and Amikacin MICs determined for isolates from Patient 1

	Clarithromycin MIC (µg/ml)					
	Day 5	Day 6	Day 8	Day 9	Day 12	Day 14
ED1-1	8	> 16	> 16	> 16	> 16	> 16
ED1-2	8	> 16	> 16	> 16	> 16	> 16
ED1-3S	8	> 16	> 16	> 16	> 16	> 16
ED1-3R	4	8	> 16	> 16	> 16	> 16
ED1-4S	8	> 16	> 16	> 16	> 16	> 16
ED1-4R	8	> 16	> 16	> 16	> 16	> 16

	Cefoxitin MIC (µg/ml)					
	Day 5	Day 6	Day 8	Day 9	Day 12	Day 14
ED1-1	32	64	64	64	64	128
ED1-2	128	128	128	128	128	128
ED1-3S	64	128	128	128	128	128
ED1-3R	128	128	128	128	128	128
ED1-4S	64	128	128	128	>128	>128
ED1-4R	128	128	128	128	>128	>128

	Amikacin MIC (µg/ml)					
	Day 5	Day 6	Day 8	Day 9	Day 12	Day 14
ED1-1	8	32	32	32	32	32
ED1-2	16	32	32	32	32	32
ED1-3S	32	32	32	32	32	32
ED1-3R	32	32	32	32	32	32
ED1-4S	8	16	16	16	32	32
ED1-4R	16	32	32	32	32	32

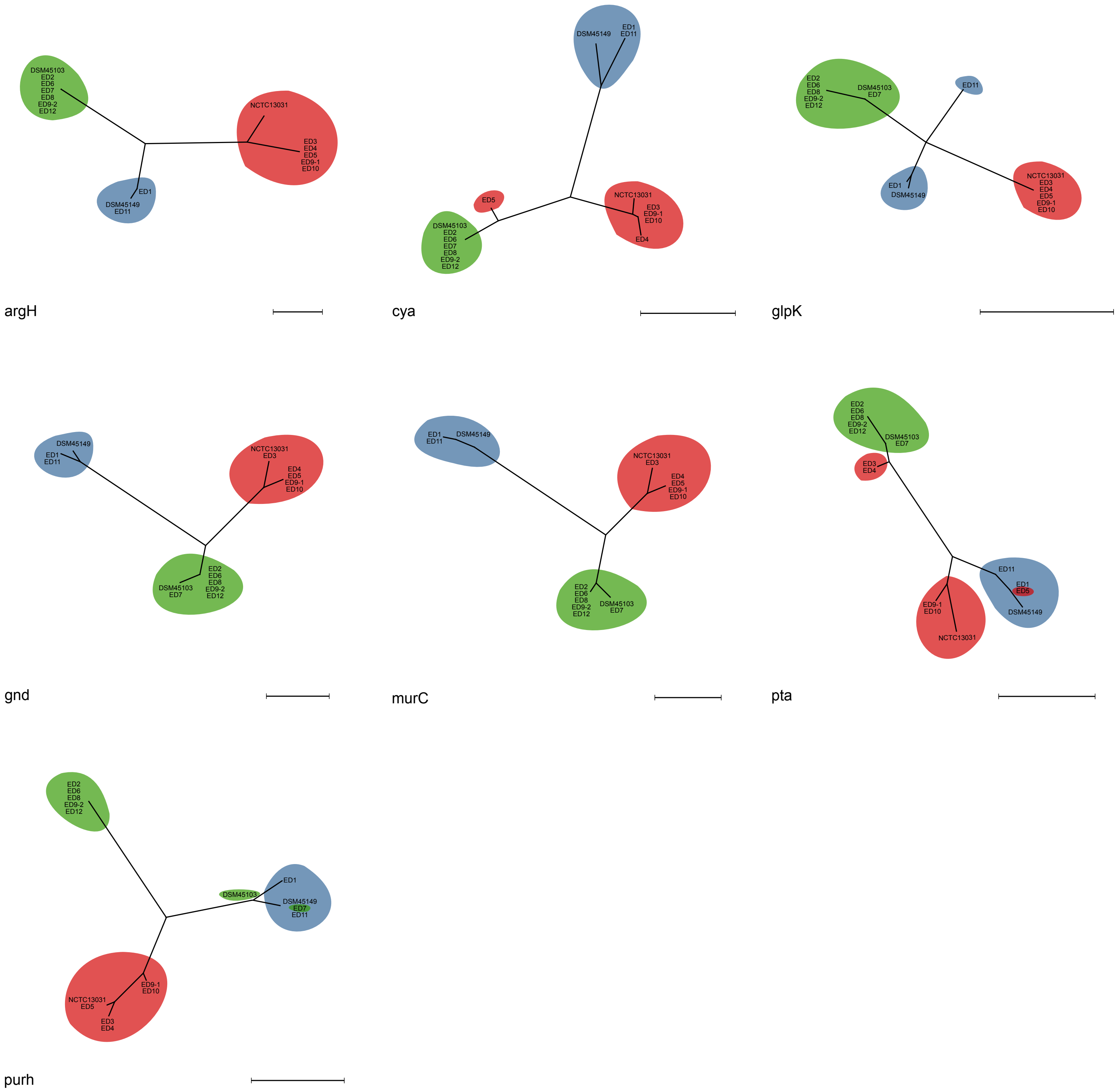

Fig. S1. Single gene trees of individual MLST alleles. Neighbour-joining phylogenies for 13 clinical isolates and type strains *M. abscessus* NCTC 13031, *M. bolletii* DSM 45149 and *M. massiliense* DSM 45103. Scale bar represents 0.01 substitutions per site. *M. abscessus*, *M. bolletii* and *M. massiliense* strains are highlighted in red, green and blue respectively.
